# A retrospective study of contributing factors for prognosis and survival length of cryptococcal meningoencephalitis in Southern part of China (1998–2013)

**DOI:** 10.1186/s12879-015-0826-y

**Published:** 2015-02-19

**Authors:** Hui Zheng, Mingyue Li, Yingting Luo, Dongmei Wang, Jialing Yang, Qiong Chen, Junying Lao, Ningfen Chen, Man Yang, Qun Wang

**Affiliations:** Department of Neurology, Nanfang Hospital, Southern Medical University, No.1838 North Guangzhou Avenue, Guangzhou City, Guangdong Province People’s republic of China; Medical Records Room, Nanfang Hospital, Southern Medical University, No.1838 North Guangzhou Avenue, Guangzhou City, Guangdong Province People’s republic of China

**Keywords:** Cryptococcal meningoencephalitis, China, Contributing factors, Survival length

## Abstract

**Background:**

Cryptococcal meningoencephalitis (CM) is the most common opportunistic infection of the central nervous system (CNS). Despite this observation, there have only been a few studies analyzing clinical characteristics as well as cerebrospinal fluid (CSF), electroencephalograph (EEG), and magnetic resonance imaging (MRI) features in CM patients of all ages.

**Methods:**

We reviewed the medical records of all patients diagnosed with cryptococcal meningoencephalitis from 1998 to 2013 in the Nanfang Hospital in China and gathered data on the underlying diseases, bird exposure history, and clinical features, including those from CSF, EEG and MRI.

**Results:**

CM is more likely to infect adults younger than 60 years old. 71.3% of CM patients were male. Unlike data from other countries, we found that chronic use of corticosteroids or other immunosuppressants (17.59%) was the most frequent risk factor in CM patients rather than HIV infection (1.85%). Clear exposure with bird/ bird droppings before CM onset is obvious in a previous study in CM children. However, our study found that 4.63% CM patients had such an exposure. More importantly, patients with brain tissue damage (p = 0.021) and decreased CSF/blood glucose ratio (p = 0.008) were significantly associated with death, but only the decreased CSF/blood glucose ratio was the contributing factor of prognosis (odds ratio, 0.047; p = 0.025). Decreased CSF/blood glucose ratio was significantly related to the survival length of CM (odds ratio, 0.134; p = 0.033).

**Conclusions:**

Our study shows that CM has predilection for young male adults. The chronic use of corticosteroids or other immunosuppressants, rather than HIV infection or bird/bird droppings exposure, was the most frequent risk factor in CM patients in our study. Decreased CSF/blood glucose ratio was both an independent contributing factor to death and was significantly related to the survival length of CM patients. The more decreased the CSF/blood glucose ratio was, the worse prognosis and shorter survival length CM patients had.

**Electronic supplementary material:**

The online version of this article (doi:10.1186/s12879-015-0826-y) contains supplementary material, which is available to authorized users.

## Background

Cryptococcus neoformans can infect almost all organs in the human body, but the infection site of serious consequence is the central nervous system (CNS) [1-3]. Cryptococcus is the most common disease causing agent of the CNS in not only HIV-positive patients and HIV-negative patients undergoing various immunosuppressive regimens, but also immunocompetent patients [4-8].

Unlike data collected from North America [9,10], South America [7,8], Europe [11] and other countries in Asia [5,6,12], the incidence of cryptococcosis in immunocompetent patients is high in China. Chen et al. [1] had summarized some features of cryptococcosis in China, but their focus was towards therapy. Guo [13], on the other hand, found that the cerebrospinal fluid (CSF) of children with cryptococcal meningoencephalitis (CM) has particular characteristics but these characteristics cannot be generalized for all other age groups. Here, we collected the epidemiological and clinical characteristics, including analysis of CSF, electroencephalograph (EEG), and magnetic resonance imaging (MRI) features of both children and adult CM patients from Nanfang Hospital. The collected data was used to analyze special characteristics of CM in the southern part of China. In addition, we attempt to elucidate the independent contributing factors for the prognosis, in hopes to streamline the decision of the appropriate CM therapy.

## Methods

### Data source and study population

Over a period from July 1, 1998 to June 30, 2013 (15 years), 108 patients from 11 months to 81 years of age at the Nanfang Hospital were retrospectively identified as having CM. Written approval for this study was obtained from the ethics committee of the Nanfang Hospital. All patients or their family members signed a written consent in accordance with the ethical committee standards during their hospital stay or outpatient follow-up. In the present study, the underlying diseases, bird exposure history, clinical features, including CSF, EEGs and MRI of CM patients were shown and the probable contributing factors for death and survival length were analyzed.

### Study criteria

CM was diagnosed by either of the following methods: (1) isolation of cryptococcus from previous or current cerebrospinal fluid cultures, followed by a positive CSF cryptococcal antibody, positive CSF India ink staining, or positive CSF Aley new blue dye staining and clinical features of meningoencephalitis; or (2) isolation of cryptococcus in blood culture with clinical presentations of meningoencephalitis and typical CSF features [10].

Patients were regarded as “with apparent risk factors” when they have HIV infection, malignancies, cirrhosis, organ transplantation, end-stage renal failure autoimmune disorder, diabetes mellitus, idiopathic CH4 T-cell lymphopenia, sarcoidosis, chronic usage of corticosteroids or other immunosuppressive therapy [14], and any abnormal status (e.g. SLE and other systemic autoimmune diseases, chronic kidney diseases, pregnant, mycosis infection of other system, drug addiction, etc.).

The MRI features of CM were diagnosed by the following standard. Cerebral ischemic stroke was defined according to the WHO criteria [15], and Bubble sign was as follows [16]: lesions frequently seen in the basal ganglia, thalami, and midbrain, hyperintensity on T2-weighted images and fluid attenuated inversion recovery (FLAIR) images without contrast enhancement.

### Therapy

The 108 patients received treatment with an intravenous administration of amphotericin B deoxycholate (AmBd), which was given at 0.1 mg/kg the first day, 0.5 mg/kg the second day and was increased to 1 mg/kg per day from the third day, together with an oral 5-fluorocytosine (5-FC) at 100 mg/kg per day and fluconazole (FCZ) at 400 mg per day, when they were diagnosed with CM. After 4–6 weeks of treatment, if cryptococcus was no longer present in at least three sequential CSF examinations by microscopy, we stopped AmB. We continued to use FCZ and 5-FC for 6–9 more months. The patients with persistent high intracranial pressure received a lumber puncture every 2 or 3 days to relieve their symptoms.

### Data analysis

Data was analyzed using SPSS 13.0 software. The categorical variables were performed by number (N)(%) and the continuous variables by mean ± standard deviation (SD). Chi-Square test was used to compare the categorical variable, including gender, age, time interval from illness onset to hospitalization, bird/ bird dropping exposure history and underlying diseases between patients recovered and those dead, while independent sample *t*-test was used to examine CSF protein, CSF glucose level and CSF/blood glucose ratio. All variables with a p-value <0.10 on Chi-Square test and *t*-test were included in a multivariate conditional logistic regression model and a Cox regression model to evaluate their independent effect and relationship to survival length. Statistical significance was defined as p < 0.05.

## Results

### The demographical characteristics

The demographical characteristics of 108 CM patients collected during 15 years in the southern part of China are shown in Table 1. Most of them were adults less than 60 years old and 15 were children (including 18 years old) (Mean age 37.40 ± 17.69 years old; Range: 11 months-81 years) (Table 1). Among the 108 patients, 77 (71.30%) were male and 31 (28.70%) were female. The mean age of male and female were 38.95 ± 18.32 years and 33.55 ± 15.65 years old, respectively. Average time interval from illness onset to hospitalization was 47.38 ± 72.33 days. 47 of 108 cases (43.52%) showed apparent risk factors. The most common risk factor was chronic use of corticosteroids or other immunosuppressants (17.59%), and systemic lupus erythematosus (SLE) was found in 8 (7.41%) cases and tuberculosis in 6 (5.56%) cases (1 with a CNS infection and 5 with lung infection). Other risk factors contain hematopathy (4.63%), diabetes mellitus (DM,4.63%), kidney diseases (3.70%), HIV infection (1.85%), organ transplantation (0.93%), nasopharyngeal carcinoma (0.93%), hyperthyroidism (0.93%), pregnant (0.93%), hepatitis B infection (0.93%), peptic ulcer (0.93%) and mycotic infection (0.93%). 5 cases (4.63%) of 108 had clear exposure with bird/ bird droppings before CM onset.

**Table 1:** **Characteristics of patients of cryptococcal meningoencephalitis**

**Characteristic**	**Recovered [no. (%)] see**	**Dead [no. (%)]**	**Lost# [no. (%)]**	**Total**	**p** ^**#**^
**Age, years**	49(45.37)	45(41.67)	14(12.96)	108	NS
0-10	2(1.85)	1(0.93)	2(1.85)	5	
11-20	7(6.48)	6(5.56)	2(1.85)	15	
21-30	6(5.56)	9(8.33)	4(3.70)	19	
31-40	13(12.04)	10(9.26)	2(1.85)	25	
41-50	8(7.41)	8(7.41)	2(1.85)	18	
51-60	10(9.26)	3(2.78)	1(0.93)	14	
61-70	2(1.85)	3(2.78)	1(0.93)	6	
71-80	1(0.93)	4(3.70)	0(0)	5	
81-	0(0)	1(0.93)	0(0)	1	
**Gender**	49(45.37)	45(41.67)	14(12.96)	108	NS
Male	36(33.33)	30(27.78)	11(10.19)	77	
Female	13(12.04)	15(13.89)	3(2.78)	31	
**Time interval from illness onset to hospitalization (days)**	49(45.37)	45(41.67)	14(12.96)	108	NS
1-10	9(8.33)	6(5.56)	1(0.93)	15	
11-20	10(9.26)	13(12.04)	3(2.78)	25	
21-30	13(12.04)	18(16.67)	5(4.63)	36	
31-	17(15.74)	8(7.41)	5(4.63)	29	
**Underlying disease** ^*^	22(20.37)	21(19.44)	4(3.70)	47	NS
**Exposure history**	2(1.85)	3(2.78)	0(0)	5	NS
**Brain tissue damaged** ^**^	9(8.33)	18(16.67)	4(3.70)	31	0.021
**Laboratory findings**					
High intracranial pressure	43(39.81)	35(32.41)	13(12.04)	91	NS
Increased CSF protein	38(35.19)	31(28.70)	10(9.26)	79	NS
Decreased CSF glucose	26(24.07)	29(26.85)	9(8.33)	64	NS
**Abnormal EEG** ^***^	16(42.11)	9(23.68)	2(5.26)	27	NS
**Abnormal MRI** ^****^	20(41.67)	10(20.83)	4(8.33)	34	NS

*Underlying diseases included use of corticosteroids or other immunosuppressants 19 cases, SLE 8 cases, Tuberculosis 6 cases (1 with CNS infection and 5 with lung infection), hematopathy 5 cases (2 with ITP, 1 with leukemia, 1 with NHL and 1 with AIHA), DM 5 cases, kidney diseases 4 cases (2 with renal failure, 1 with nephrotic syndrome and 1 with RPGN), HIV 2 cases, organ (renal) transplantation 1 case, cancer (nasopharyngeal carcinoma) 1 case, hyperthyroidism 1 case, pregnant 1 case, hepatitis B 1 case and mycotic infection 1 case.

**Brain tissue damage included altered consciousness 17 cases, seizures 6 cases, cerebellum symptoms 6 cases and mental disorders 4 cases. (Several cases with more than one symptom).

***Abnormal EEG included normal background 20 cases (1 with epileptiform discharge and 19 with focal slow wave) and diffused slow wave in the background 7 cases.

****Abnormal MRI included Bubble sign 9 cases (6 at the basal ganglion and 3 at subcortex), hydrocephalus 16 cases, abnormal lesions 16 cases (8 cases with hemilateral lesions, 8 cases with bilateral lesions) and ischemic lesions 3 cases (2 with ischemic lesion and 1 with transient ischemic attack) (most cases with more than one abnormal features).

^**#**^NS, not significant (p > 0.05).

### Clinical features

Nonspecific symptoms, such as headache (75.00%) and fever (62.04%) were frequently found in CM patients. However, symptoms associated with brain tissue damage (17 with unconsciousness, 6 with seizures, 6 with cerebellum symptoms and 4 with psychosis) were also seen (Table 1).

We collected the first CSF results of these 108 CM patients, and found that increased intracranial pressure was the most common change (84.26%), including 72 cases higher than 30 cm H_2_O (seriously abnormal). Increased protein, decreased glucose, and decreased CSF/blood glucose ratio were also apparent in CSF of CM patients (Table 1).

38 (35.19%) of the 108 CM patients had EEG examinations in our hospital and 11 (28.95%) were normal. Among the abnormal EEG results, 1 (2.63%) had epileptiform discharge, 7 (18.42%) with slow wave background and 19 (50.00%) had normal background with diffused slow wave.

48 (44.44%) patients had cranial MRI in or out of our hospital and 34 of them had abnormal features. 9 (18.75%) of these cases had Bubble sign, 6 (12.50%) of which were at basal ganglions and 3 (6.25%) at subcortex. 16 (33.33%) of the 48 cases had abnormal lesions at brain hemisphere, including 8 (16.67%) bilateral and 8 (16.67%) hemilateral. In addition, 16 (33.33%) cases had hydrocephalus and 3 patients (6.25%) had ischemic lesions.

### Outcome

The median length of hospitalization was 41 days (range: 1–389 days), and the median survival length was 4.51 months (range: 0.03-160.68 months). According to the follow up on Aug, 2013, 49 (45.37%) cases were recovered, 45 (41.67%) cases were dead of CM and 14 (12.96%) cases were lost due to discontinued communication (Table 1). The survival patients were scored according to the modified Rankin Scale (mRS) when they came to the hospital, and the latest mRS score of them were shown in Table 2. 14 patients had no symptoms at all; 21 mild symptoms or slight disability (10 scored 1 and 11 scored 2); 12 moderate disability (9 scored 3 and 3 patients scored 4), while 6 severe disability.

**Table 2:** **mRS score of survival patients of cryptococcal meningoencephalitis**

**mRS score**	**Number of patients**
**0**	14
**1**	10
**2**	11
**3**	9
**4**	3
**5**	6
**6**	45
**Censored**	14

### Contributing factors of death and survival length

Chi-Square test and independent sample *t*-test were used to identify factors related to prognosis (Tables 1 and 3), which indicated that patients with brain tissue damage (p = 0.021) and decreased

**Table 3:** **Features of CSF**

	**Recovered**	**Dead**	**p** ^**#**^
**Ratio of CSF glucose/blood glucose**	0.3426 ± 0.16447	0.246 ± 0.17048	0.008
**CSF glucose**	2.1439 ± 1.13714	1.8089 ± 1.44314	NS
**CSF protein**	0.7937 ± 0.55197	0.7609 ± 0.50626	NS

^**#**^NS, not significant (p > 0.05).

CSF/blood glucose ratio (p = 0.008) were significantly associated with death. Then multivariate logistic analysis was performed to determine the independent contributing factors for death of CM (Table 4). Compared to patients with normal CSF/blood glucose ratio (1/2-2/3), others with decreased CSF/blood glucose ratio were more likely to die than recover (Odds ratio(OR) = 0.047; 95%CI =0.003-0.685; p = 0.025).

**Table 4:** **Multivariate conditional logistic analysis of risk factors for prognosis of cryptococcal meningoencephalitis patients**

**Risk factors**	**p** ^**#**^	**OR (95% CI)**
**Decreased CSF glucose/blood glucose ratio**	0.025	0.047(0.003-0.685)
**With Brain tissue damaged symptoms**	NS	2.252(0.841-6.032)

^**#**^NS, not significant (p > 0.05).

Furthermore, we made use of Cox regression analysis of CM patients (Table 5) and found decreased CSF/blood glucose ratio was significantly related to the decreasing survival length of CM patients(OR = 0.134; 95%CI = 0.021-0.847; p = 0.033).

**Table 5:** **Cox regression analysis of risk factors for survival length of cryptococcal meningoencephalitis patients**

**Risk factors**	**p** ^**#**^	**OR (95% CI)**
**The ratio of CSF glucose/blood glucose**	0.033	0.134(0.021-0.847)
**With brain tissue damaged symptoms**	NS	1.562(0.829-2.942)

^**#**^NS, not significant (p > 0.05).

In summary, we demonstrated that decreased CSF/blood glucose ratio and having brain tissue damage symptoms were associated with prognosis in CM patients, but only decreased CSF/blood glucose ratio was an independent contributing factor. Furthermore, CSF/blood glucose ratio was significantly related to the survival length of CM. The more decreased the CSF/blood glucose ratio was, the worse prognosis and shorter survival length CM patients had.

## Discussion

In this study, we analyzed 108 patients diagnosed with CM from Nanfang Hospital in the southern part of China and identified some characteristics of CM from them. Although CM may affect people of any age [17], neonates, children and the elderly were not the predilection in our study, mostly because of their limited environmental exposure. In accordance with other studies [12], male predominance (71.30%) was apparent in this study, the reason of which was not fully understood, but can be due to increased environmental exposure, hormonal effect and/or genetic predisposition [3].

Dramatically lower than in Malaysia (37.5%) [12], Europe (68%) [7] and United States (89%) [4], 2 cases (1.85%) were HIV positive in CM patients in our study. Nevertheless, chronic use of corticosteroids or other immunosuppressants (17.59%) was the most common risk factor in Chinese CM patients, probably as a result of the increasing morbidity of autoimmune disease and less attention of side effects of such drugs from patients and care givers. Unlike the exposure history in HIV negative children [13], there was fewer people who had bird/bird dropping exposure in adults with CM.

Among nonspecific symptoms, the most common symptoms were lower than reported by a previous study by Chen, with obvious differences in headache (75.00% vs. 90%) and fever (62.04% vs. 90%) [1]. Symptoms associated with brain tissue damage, like unconsciousness, seizures, cerebellum symptoms and psychosis were fewer, which demonstrated the preferred location of CM was meninges rather than brain parenchyma. Furthermore, cryptococcosis should be taken into consideration when patients present with prolonged headache [18], whether fever exists or not.

Most of the CM patients had abnormal EEG, but the EEG results could not directly be attributed to diagnosis of CM.

MRI had some features indicating probable cryptococcal meningoencephalitis, which should be of particular interest. The common imaging features of cryptococcal meningoencephalitis reported previously included non-enhancing dilated Virchow-Robin space, gelatinous pseudocysts (bubble signs), leptomeningeal enhancement, hydrocephalus, cyptococcoma, hemorrhagic infarction, and abscess [18-20]. In our study, however, gelatinous pseudocysts (Bubble signs) and hydrocephalus were the common ones. Although gelatinous pseudocysts (18.75%) - which were symmetrically hyperintense on T2-weighted and FLAIR images in the basal ganglia, thalami and midbrain - did not appear in each case, its appearance strongly pointed to cryptococcus spreading along the Virchow–Robin spaces (VRS) [16]. Hydrocephalus is another characteristic of cryptococcal meningoencephalitis, but only one third (33.33%) of patients with MRI in this research presented this characteristic, probably due to the early appearing high intracranial pressure.

Cerebrospinal fluid is the most important examination in the diagnosis of CNS infections including CM. Although previous studies [18,19] mentioned increased CSF pressure, increased protein and decreased glucose level of the CSF, there has not been any detailed descriptions about their changes. In our study, about 84.26% patients had high intracranial pressure, 79.12% of which were higher than 30 cm H_2_O. So far, there is no pathophysiologic mechanisms about the change, but it can be linked to impaired CSF reabsorption as a consequence of a direct cryptococcal infiltration of the villi [21]. Reduced CSF outflow possibly resulting from an increased outflow resistance - though not necessarily accompanied by prominent cerebral edema [22] - can to some extent explain the ICP in cryptococcal meningoencephalitis. Interestingly, from our analysis, patients with high CSF pressure did not correlate to worse prognosis, as previously hypothesized, probably because of a longer hospitalization period resulting in increased attention from doctors and family members. As a result of increased protein secretion stimulated by cryptococcus and blockage of CSF circulation, 73.15% of 108 patients had increased CSF protein and 41.77% were more than 1 g/L. 64 cases (59.26%) of the 108 patients had decreased CSF glucose level, 32 of which had lower than 1 mmol/L. In addition, patients with lower CSF glucose level often had worse prognosis, probably because low CSF glucose usually indicated high cryptococcus titer and weak host immunity state. This mechanism can be our future work of the CM study.

Darze [23] reported that consciousness level contributes to CM prognosis, while other studies demonstrated a lower CSF opening pressure and a change of white blood cell in CSF was associated to a higher lethality rate [24]. In our study, however, we analyzed both symptoms induced by brain tissue damage, such as change of consciousness level, epilepsy, cerebellum symptoms and psychosis, and CSF changes in CM patients. We found that symptoms induced by brain tissue damage were associated with prognosis, but not an independent contributing factor. The decreased CSF/blood glucose ratio was an independent contributing factor for prognosis instead of a low CSF glucose level, a change of CSF pressure, or a change of white blood cells in CSF,. Patients with decreased CSF/blood glucose ratio were unlikely to recover and inclined to die. Furthermore, CM patients with decreased CSF/blood glucose ratio had significantly shorter survival length.

Although our study firstly identified the valuable contributing factors for death and survival length of CM patients, there were some limitations in it. We followed up with most of the CM patients, but some cases were lost in the rural area and mountain region, which were difficult to either reach or contact by phone or mail. Probably due to this lost data, the result presented in our survival curve (see Additional file 1) could not effectively portray the changes between patients with decreased CSF/blood glucose ratio and those with a normal ratio. Improvement of follow-up methods may give us more information. Scarcity of EEG and cranial MRI data collected limited the potential of finding valuable clues of CM. In addition, a larger sample size used in our study may allow the probability to detect more contributing factors for mortality and survival length, besides decreased CSF/blood glucose ratio and symptoms from brain tissue damage. Continuous surveillance or a multicenter study should be taken for a more detailed study.

## Conclusions

CM is the most opportunistic infection of the CNS worldwide. Our study showed some characteristics of CM in the southern part of China. CM is more likely to infect young adult rather than children and elderly, and more likely to infect male rather than female. Chronic use of corticosteroids or other immunosuppressants, rather than HIV infection, was the most frequent risk factor in CM patients in our study. Bird or bird dropping exposure was not as important in the whole population as in children. Decreased CSF/blood glucose ratio and presence of brain tissue damage symptoms were associated with CM, but only decreased CSF/blood glucose ratio was an independent contributing factor. Furthermore, CSF/blood glucose ratio was significantly related to the survival length of CM patients. The more decreased the CSF/blood glucose ratio was, the worse prognosis and shorter survival length CM patients had.

## Additional file

Additional file 1:
**The survival curve of cryptococcal meningoencephalitis patients.**

## Abbreviations

CM: Cryptococcal meningoencephalitis
CNS: Central nervous system
CSF: Cerebrospinal fluid
EEG: Electroencephalograph
MRI: Magnetic Resonance Imaging
OR: Odds ratio
CI: Confidence interval
FLAIR: Fluid attenuated inversion recovery
AmBd: Amphotericin B deoxycholate
5-FC: 5-fluorocytosine
FCZ: Fluconazole
SD: Standard deviation
SLE: Systemic lupus erythematosus
