# Supplementary material for: A retrospective study of contributing factors for prognosis and survival length of cryptococcal meningoencephalitis in Southern part of China (1998–2013)
Source: BMC Infect Dis. 2015 Feb 19;15:77. doi: 10.1186/s12879-015-0826-y (PMC4349236; doi:10.1186/s12879-015-0826-y)
Supplement: Supplementary file 1 [file 12879_2015_826_MOESM1_ESM.doc]

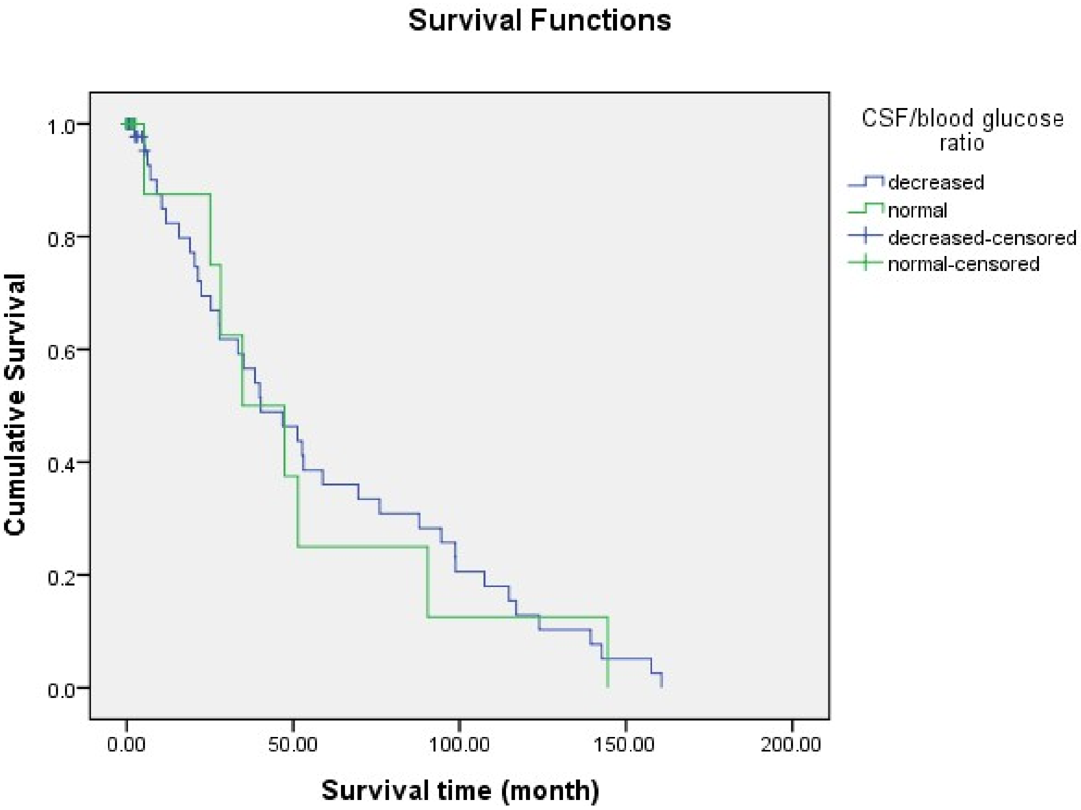

Survival Functions
Cumulative Survival
1.0
0.8
0.6
0.4
0.2
0.0
0.00
50.00
100.00
150.00
200.00
Survival time (month)
CSF/blood glucose ratio
decreased
normal
decreased-censored
normal-censored
